# Successful Whole Genome Nanopore Sequencing of Swine Influenza A Virus (swIAV) Directly from Oral Fluids Collected in Polish Pig Herds

**DOI:** 10.3390/v15020435

**Published:** 2023-02-04

**Authors:** Nick Vereecke, Aleksandra Woźniak, Marthe Pauwels, Sieglinde Coppens, Hans Nauwynck, Piotr Cybulski, Sebastiaan Theuns, Tomasz Stadejek

**Affiliations:** 1 Laboratory of Virology, Faculty of Veterinary Medicine, Ghent University, 9820 Merelbeke, Belgium; 2 PathoSense BV, 2500 Lier, Belgium; 3 Department of Pathology and Veterinary Diagnostic, Institute of Veterinary Medicine, Warsaw University of Life Sciences-SGGW, 02-776 Warsaw, Poland; 4 Goodvalley Agro S.A., Dworcowa 25, 77-320 Przechlewo, Poland

**Keywords:** epidemiology, nanopore sequencing, sample storage, viral genomics, surveillance

## Abstract

Influenza A virus (IAV) is a single-stranded, negative-sense RNA virus and a common cause of seasonal flu in humans. Its genome comprises eight RNA segments that facilitate reassortment, resulting in a great variety of IAV strains. To study these processes, the genetic code of each segment should be unraveled. Fortunately, new third-generation sequencing approaches allow for cost-efficient sequencing of IAV segments. Sequencing success depends on various factors, including proper sample storage and processing. Hence, this work focused on the effect of storage of oral fluids and swIAV sequencing. Oral fluids (n = 13) from 2017 were stored at −22 °C and later transferred to −80 °C. Other samples (n = 21) were immediately stored at −80 °C. A reverse transcription quantitative PCR (RT-qPCR) pre- and post-storage was conducted to assess IAV viral loads. Next, samples were subjected to two IAV long-read nanopore sequencing methods to evaluate success in this complex matrix. A significant storage-associated loss of swIAV loads was observed. Still, a total of 17 complete and 6 near-complete Polish swIAV genomes were obtained. Genotype T, (H1avN2, seven herds), P (H1N1pdm09, two herds), U (H1avN1, three herds), and A (H1avN1, 1 herd) were circulated on Polish farms. In conclusion, oral fluids can be used for long-read swIAV sequencing when considering appropriate storage and segment amplification protocols, which allows us to monitor swIAV in an animal-friendly and cost-efficient manner.

## 1. Introduction

Swine influenza A virus (swIAV) causes respiratory disease in pigs, of which the clinical picture varies from subclinical to severe [1]. The virus’ genome comprises eight segments of single-stranded, negative-sense RNA. In general, IAV strains are typed according to their surface glycoproteins, hemagglutinin (HA) and neuraminidase (NA), which are encoded by segments 4 and 6, respectively. The remaining segments are referred to as the internal gene cassette (IGC). Such a structure of the IAV genome allows for the exchange of the segments, also known as reassortment, if two or more viruses infect a single cell. This can eventually result in the emergence of new swIAV strains [2]. The lack of exonuclease proofreading of the virus’ RNA polymerase results in an increased mutational rate (mean of 2.3 × 10^−5^ substitutions per nucleotide per cell infection for the whole genome [3]), promoting genetic drift. These, together with the fact that many IAV strains can be transmitted between different species, led to the complex and ever-changing picture of swIAV genetic and antigenic diversity across the world.

Currently, three major swIAV subtypes circulate in swine in Europe, including H1N1, H3N2, and H1N2. However, the origin of HA- and NA-encoding segments may differ between the strains from the same subtype, which makes actual swIAV subtyping even more complex. To address the highly complex picture of currently circulating swIAV reassortment, a first global swine H1 nomenclature system was introduced by Anderson and colleagues in 2010. Their aim was to classify swIAV strains based on their HA gene sequence into a classical lineage (1A), human seasonal lineage (1B), and Eurasian avian lineage (1C) [4]. Later, Watson et al. (2015) proposed swIAV genotype classification based on the assignment of each segment to one of nine genetic lineages: (i) Eurasian avian-like H1avN1; (ii) A/swine/Gent/1/1984-like H3N2; (iii) A/swine/Scotland/410440/1994-like H1huN2; (iv) A/swine/Italy/4675/2003-like rH1N2; (v) North American triple reassortment; (vi) classical H1N1; (vii) A(H1N1)pdm09; (viii) human seasonal H3N2; and (ix) avian. The analysis of 290 viruses isolated between 2009 and 2013 identified 23 distinct combinations of segments which determined genotypes A through W in Europe [5]. Later, Henritzi et al. (2020) identified as many as 31 genotypes (with the addition of AA-AP) among 233 viruses isolated in Europe from 2015 to 2018 [6].

Precise swIAV subtyping and genotype assignment is possible by using reverse transcription quantitative PCR (RT-qPCR) [7] or nucleotide sequencing. Recently, different next-generation sequencing (NGS) methods have been more widely used, especially for whole-genome sequencing (WGS). Of these, Illumina short-read sequencing is the most frequently used, and more recently, long-read, third-generation alternatives (e.g., Oxford Nanopore Technologies (ONT)) have also been introduced into IAV genomics and diagnostics [8].

Whole-genome sequencing of swIAV is most often performed on virus isolates, but Illumina sequencing directly from nasal swabs was also reported [9]. For successful virus isolation, nasal swab samples or respiratory tract tissue must be collected from acutely infected animals in order to contain sufficiently high virus loads. However, the identification of such animals can be difficult in cases of a mild course of influenza (i.e., endemic infections), which is not uncommon. Henritzi et al. (2020) reported that only 30.5% of 18,313 nasal swabs from pigs with respiratory disease from European countries were positive, based on the generic matrix (M) gene-specific qPCR [6]. The proportion of positive samples differed greatly between countries. For example, 41.6% of 4064 clinical samples from Germany were found with RT-qPCR to be positive, whereas in Poland only 12.8% of a total of 524 samples were positive. It is unknown whether this discrepancy can be attributed to misdiagnosis due to wrong timing or swabbing technique, the improper handling of samples (e.g., storage prior to testing), or differences in influenza prevalence between these countries, which likely exist.

Recently, oral fluids have been frequently used as samples for routine PCR-based diagnostics, monitoring, and surveillance of multiple swine viruses and bacteria, such as porcine reproductive and respiratory syndrome virus (PRRSV), porcine circovirus type 2 and 3 (PCV2 and PCV3), and swIAV, as well as bacteria such as *Lawsonia intracellularis* and *Brachyspira* spp. [10,11,12,13]. Oral fluids can be considered a collective sample that represents a pen of pigs. Unlike individual blood or nasal swab samples, which are usually limited in number and collected per herd, oral fluid collection can assist in the simultaneous sampling of multiple pens and age groups (populations). This facilitates the detection of pathogens at the early stage of infection in convalescent animals or in subclinically infected populations. However, it is important to position oral fluid ropes in a correct way (i.e., by height, animal density, etc.) to draw proper conclusions [10].

Several studies described IAV detection in oral fluids [10,11]. Decorte et al. (2015) reported the detection of IAV RNA at 21 days post-infection in 25% of oral fluid samples, while nasal swabs reacted negatively seven days post-infection [10]. This prolonged detection of IAV in oral fluids can be explained by the detection of viral RNA in expectorated sputum, which contains cellular debris from the lower respiratory tract. Extended periods of swIAV detectability in oral fluids may potentially facilitate the detection of swIAV outbreaks, especially those with a mild or subclinical course (i.e., endemic infections). However, the complex nature of these oral fluid samples, which contain oral mucins, proteolytic and nucleolytic enzymes, drug components, food particles, and fecal material, makes it a difficult matrix for the detection of viruses, especially for RNA targets [14]. Therefore, the samples should be chilled and/or frozen immediately after collection, and nucleic acid extraction should be optimized [15].

Targeted NGS has been performed for PRRSV, porcine astrovirus, and PCV3 with oral fluids [16,17,18,19]. With the availability of third-generation sequencing technologies (e.g., ONT), swIAV seems an ideal subject for targeted WGS from oral fluid samples. This could significantly facilitate the surveillance of the genetic diversity of this ever-changing porcine virus. It must be stressed that from many countries, the information on the current situation of the prevalence of swIAV genotypes is missing. For Polish swIAV, only 21 sequences of HA and 23 of NA segments are currently available in public repositories.

In this work, we aimed at filling the gap in the knowledge on Polish swIAV genomic diversity. To carry out this research, we applied nanopore long-read sequencing to archived oral fluid samples obtained from Polish herds between 2017 and 2020. Moreover, we showed the importance of oral fluid storage conditions for successful WGS of the swIAV virus and compared two protocols for swIAV segment amplification for subsequent nanopore sequencing.

## 2. Materials and Methods

### 2.1. Collection of Oral Fluids from Polish Herds

Oral fluid samples were collected from 2017 to 2020 from Polish pig farms representing different sizes and types of production. Samples were collected from pigs of different ages that were exhibiting influenza-like clinical signals, as identified by the farm veterinarian. The samples were obtained as described previously, chilled, and transported to the laboratory in order to minimize the impact of adverse conditions during sample handling and transport [20]. Upon delivery to the laboratory, their quality was visually assessed (e.g., color, transparency, and sediment), aliquoted, and stored either at −22 °C or −80 °C. The overview of the samples and associated metadata can be found in Supplementary Table S1.

### 2.2. Assessment of swIAV Detection Using RT-qPCR Pre- and Post-Storage

Prior to aliquoted storage, each sample was subjected to RNA isolation using the QIAamp cador Pathogen Mini Kit (Qiagen, Hilden, Germany) or IndiSPIN Pathogen Kit (Indical Bioscience GmbH, Leipzig, Germany) according to the manufacturers’ instructions. Extracted RNA was used for RT-qPCR with the virotype Influenza A RT-PCR Kit (Indical Bioscience GmbH, Leipzig, Germany) in the Rotor-Gene Q 5PLEX platform (Qiagen, Hilden, Germany). Post-storage, nucleic acids of all the oral fluid samples were extracted again using the Quick-DNA/RNA Viral Kit (Zymo Research, CA, USA). In short, viral RNA was extracted according to the manufacturers’ instructions, with the exception that an input of 400 µL and an elution in 35 µL elution buffer was applied. The RNA was subjected to a pan-IAV RT-qPCR assay as described by Hoffman and colleagues [21]. For each sample, duplicate technical replicates were included in the assay, which was run on the StepOne™ Real-Time PCR System (Applied Biosystems™, Waltham, MA, USA). Means of technical duplicates were used to assess the effect of storage conditions as represented by the difference in Cq values (ΔCq), which was obtained by subtracting pre-storage Cq values from post-storage Cq values.

### 2.3. Evaluation of Two IAV Sequencing Protocols for Oral Fluids

The post-storage RNA extracts were also used for target enrichment using two IAV whole-genome sequencing protocols, prior to long-read nanopore library preparation and sequencing on a R9.4.1 flow cell and GridION sequencer (ONT). For each method, a one-step RT-PCR was performed using the SuperScript™ III One-Step RT-PCR System with the Platinum™ Taq DNA Polymerase (Invitrogen™, Waltham, MA, USA), using 5 µL of RNA as the template. To amplify each segment of IAV, method 1 used two primers (Pan-IVA-1F_M13F/Pan-IVA-1R_M13R), as described by King et al. (2020), which were used in a simple RT-PCR protocol [22]. In method 2, three primers (CommonA-Uni12G/CommonA-Uni12/CommonA-Uni13G), as described by Van Poelvoorde et al. (2021), were applied in a complex RT-PCR. Their RT-PCR reaction differed in the RT step and included ramping rates during the five first PCR cycles [23]. The RT-PCR products were evaluated via 2% agarose gel electrophoresis and subjected to PCR clean up using CleanNGS (CleanNA, Waddinxveen, The Netherlands) beads in a 1:1 ratio. Prior to library preparation, concentrations were determined using the QuantiFluor^®^ ONE dsDNA kit (Promega, Madison, WI, USA) on the QuantiFluor^®^ (Promega, Madison, WI, USA)) device. Long-read libraries were prepared using the ligation sequencing and native barcoding kits (SQK-LSK109 and SQK-NBD96, respectively; ONT) according to the manufacturer’s instructions.

### 2.4. Genome Assembly and Epidemiological Analysis of Polish swIAV Segment Sequences

Raw sequencing data were base called using the “super accurate” base-calling model in Guppy (v6.2.7; ONT). Adapter trimming and quality filtering was performed using NanoFilt (v2.6.0; [24]). Additional primers were removed using cutadapt (v2.8; [25]). Reads were binned according to their gene segment using minimap2 (v2.17; [26]). For each segment, the corresponding read bin was used to perform *de novo* segment assembly using Canu (v2.2; [27]), minimap2, and medaka polishing (v1.4.1; ONT). A minimum depth of 30× was required. Sequences from segment 4 (HA) and 6 (NA) were extracted from complete swIAV genomes (n = 19) for subsequent downstream multiple-sequence alignment (MAFFT; v.7.453 [28]) and phylogenetic inference (IQ-tree2; -bb 1000; v.2.2.0 [29]). Sequences from this study were supplemented with 28 relevant (and ancient) reference sequences of swine and human IAVs to allow for proper clade distinction [5,30]. Additionally, 20 recent (2019–2021) isolates from Belgium and the Netherlands and the 12 available Polish sequences were added [7,31]. Clade determination of H1 segments was performed based on the global swine H1 nomenclature system by Anderson et al. (2010) [4]. Final tree visualizations were carried out using iTOL (v.6; [32]), only showing bootstrap support <95%. Lineage determination of the other segments was performed in the same way using MAFFT and reference strains as described by Chepkwony and colleagues (2021) [30]. For each of these alignments, a maximum likelihood phylogenetic tree was inferred with IQ-Tree2 (-bb 1000). Estimated segment origin was based on the closest related gene segments. Genotyping was also performed based on the classification system provided by Watson et al. (2015) [5]. If samples had one or more missing internal genes, lineage was assigned based on the available genes of the IGC. Segment sequences of complete genomes were submitted to NCBI. An overview of the accession numbers is provided in Supplementary Table S1.

### 2.5. Statistical Analysis

Statistical analyses were performed in Graphpad Prism v.9.4.1 using a nonparametric Wilcoxon matched-pairs signed-rank test with a 0.05 significance level cut-off for *p*-values.

## 3. Results

### 3.1. Impact of Storage Temperature, Time, and Oral Fluid Sample Quality on swIAV Detection

**Figure 1:**
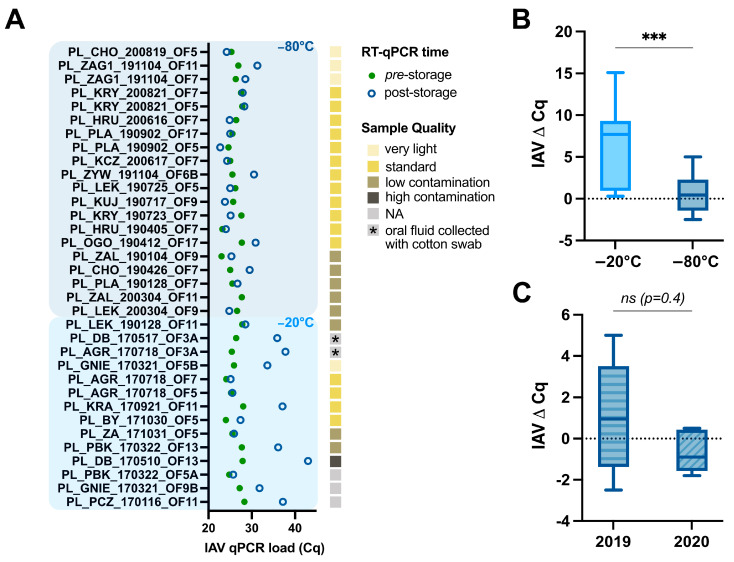
Impact of storage temperature, storage time, and oral fluid sample quality on IAV RT-qPCR detection. (**A**) Overview of IAV RT-qPCR detection of 34 oral fluid samples collected from 19 different Polish farms collected between 2017 and 2020. Samples have been categorized by their original storage temperature (−22 °C and −80 °C shaded in light and dark blue, respectively). Differences in detection (RT-qPCR) for IAV are shown as Cq values pre-storage (solid green circles) and post-storage (open blue circles). Visual inspection of sample qualities is represented as yellow-to-brown shaded boxes for each sample. NA indicates no data were available. (**B**) Box-and-whisker representation (min. to max.) of all samples as categorized based on initial storage temperature, including 13 and 21 samples for the −22 °C and −80 °C groups, respectively. (**C**) Box-and-whisker representation (min. to max.) of all −80 °C-stored samples (n = 21) further categorized by year of collection and storage. Seven and fourteen samples originated from 2019 and 2020, respectively. Statistical analyses were performed using a nonparametric Wilcoxon matched-pairs signed-rank test with a *p* < 0.05 significance cut-off; *** *p* < 0.001, ns = non significant.

A total of 34 oral fluids were collected from 19 Polish farms between 2017 and 2020 (Supplementary Table S1). While samples collected in 2017 (n = 13) were initially stored at −22 °C and transferred to −80 °C two years later, 21 samples collected between 2019 and 2020 were immediately stored at −80 °C. Prior to aliquoted storage, each sample was subjected to RNA isolation and RT-qPCR to assess its viral load (Figure 1A, solid green dots). Next, in 2022, samples were again subjected to RNA isolation, RT-qPCR, and targeted IAV WGS to assess the impact of storage on swIAV detection (i.e., Cq values pre- and post-storage) and determine the success of the IAV sequencing of oral fluids (Figure 1A, open blue circles). As summarized in Figure 1A, a clear right-handed shift in swIAV detection was observed for most (9/13) of the samples that were initially stored at −22 °C. No apparent correlations were found between oral fluid sample quality and swIAV detection, as represented by the yellow-to-brown shaded boxes in Figure 1A. However, one sample was classified as “high contamination” based on color and turbidity, and showed the highest difference in swIAV RT-qPCR detection (ΔCq = 15.1 for sample A/swine/Poland/DB_170517_OF13/2017). Interestingly, a significant ΔCq (*p* = 0.0007) was observed for samples that were stored at different temperatures. A ΔCq of 6.3 (±4.9 SD) and 0.6 (±2.3 SD) was obtained for samples stored at −22 °C and −80 °C, respectively (Figure 1B). To further assess the potential impact of prolonged (long-term) storage periods, all samples stored at −80 °C were further categorized based on their collection and storage year. Even though older samples (2019) showed a bigger standard deviation (SD) (ΔCq = 1.2 (±2.5 SD) with n = 14) in swIAV detection differences, no significant impact of longer storage was observed as compared to more recent samples (2020; ΔCq = −0.7 (±1.0 SD) with n = 6) (Figure 1C).

### 3.2. Impact of IAV Sequencing Protocol on Sequencing Success of the Eight IAV Gene Segments

**Figure 2:**
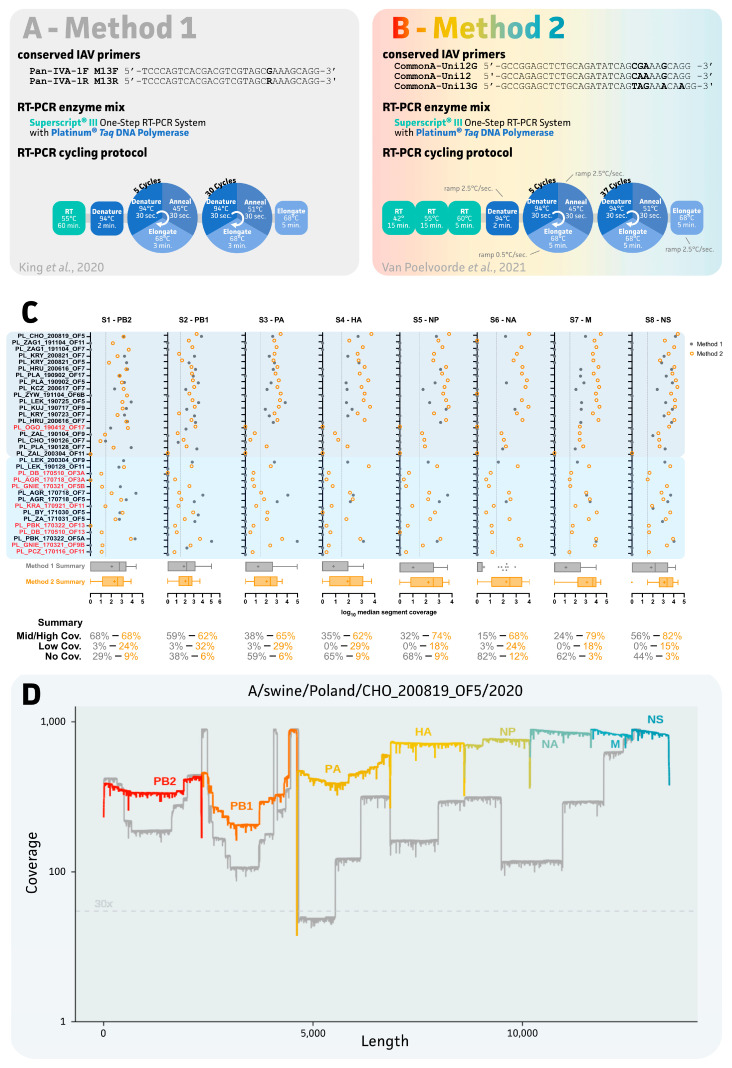
Comparative overview of sequencing procedures and swIAV segment coverage for two sequencing protocols. (**A**,**B**) Schematic representation of two sequencing protocols that were compared in this study. (**C**) Impact on median segment coverage for each sample as represented by solid grey circles and open orange circles for method 1 and method 2, respectively. Sample names were colored according to RT-qPCR detection, where red indicates invalid (Cq > 31) samples for sequencing. For sample A/swine/Poland/LEK_200304_OF9/2020, no sequencing data were available for method 2. Box-and-whisker representation (Tukey) shows overall division and mean (+) of each method. (**D**) Example of coverage plot of concatenated swIAV segments (S1 up to S8) for a selected sample (A/swine/Poland/CHO_200819_OF5/2020), which was collected in 2020 and immediately stored at −80 °C. Grey and colored coverage plots represent segment-specific coverages for sequencing method 1 and 2, respectively. Note, sequencing coverage is represented on a logarithmic (log_10_) scale on the both x- and y-axis. A dotted line was drawn to indicate the minimal required 30× sequencing coverage.

As summarized in Figure 2A,B, different conserved IAV primer sets were applied in the same one-step RT-qPCR enzymatic reaction using the SuperScript™ III One-Step RT-PCR System with the Platinum™ *Taq* DNA Polymerase (Invitrogen™, Waltham, MA, USA). Both methods used different universal primers with degenerate bases during the consecutive RT-qPCR thermocycling conditions. The second method exploited a three-step RT procedure and ramping times during the first PCR cycling (Figure 2B). As exemplified by the targeted sequencing of other viruses (e.g., SARS-CoV−2), sequencing success is highly dependent on the viral load (Cq values) within the sample (report on SeqCOVID by García Marín, 2020). Based on post-storage Cq values, 9 out of 34 samples were considered less valid for sequencing as they showed a post-storage Cq > 31, which is associated with an average reduction in swIAV detection of 7.3 (±3.4 SD) (Figure 2C sample colors and Supplementary Table S1).

Even though some samples were considered less useful for whole-genome sequencing, all samples were subjected to sequencing with both methods since initial sequencing (method 1) was initiated based on pre-storage Cq values. As summarized in Figure 2C, a clear impact on sequencing success for all IAV segments was observed when using method 2. While comparable success rates for medium/high coverage samples were observed for PB2 (S1) and PB1 (S2), ranging from 59% up to 68%, bigger differences were observed for all other segments. Method 2 showed a minimum of a two-fold increase in the sequencing success rate for each of the six remaining segments as compared to method 1. Success rate improvements of 38% > 65% (S3), 35% > 62% (S4), 32% > 74% (S5), 15% > 68% (S6), 24% > 79% (S7), and 56% > 82% (S8) were observed for segments S3 up to S8, respectively. Interestingly, sequencing success for the NA (S6) segment was three times higher using method 2 as compared to method 1. Additionally, samples lacking sequencing coverage using method 1 resulted in low-coverage sequence data for most of the eight segments with method 2 (Figure 2C). In the end, method 2 allowed us to generate a total of 14 (66.7% success rate) and 3 (23.1% success rate) complete genomes for the samples that were stored at −80 °C and −22 °C, respectively. Additionally, both methods resulted in two near-complete genomes (lacking a max. of two segments). Unfortunately, these near-complete genomes showed lower sequence coverage for one or both HA and NA segments. As expected, samples that were initially stored at −22 °C showed a higher number (8/13 or 61.5%) of low-coverage sequencing results (missing more than two genome segments) as compared to only 4 out of 21 samples (19.0%) for those immediately stored at −80 °C. Samples showing an overall high (complete) or medium/low (near-complete/low-coverage) sequencing coverage showed a mean post-storage Cq value of 25.6 (±1.7 SD) and 29.3 (±2.5 SD), respectively. Interestingly, near-complete and low-coverage genomes showed higher ΔCq values (4.0 (±1.0 SD)) as compared to −0.3 (±1.3 SD) for the complete genomes. Here, again, no clear correlation between oral fluid sample quality could be drawn (data not shown). In summary, method 1 resulted in an overall sequencing success of only 32% (8/25) as compared to 68% (17/25) for method 2.

### 3.3. Genetic Diversity of swIAV Strains in Polish Farms between 2017 and 2020

**Figure 3:**
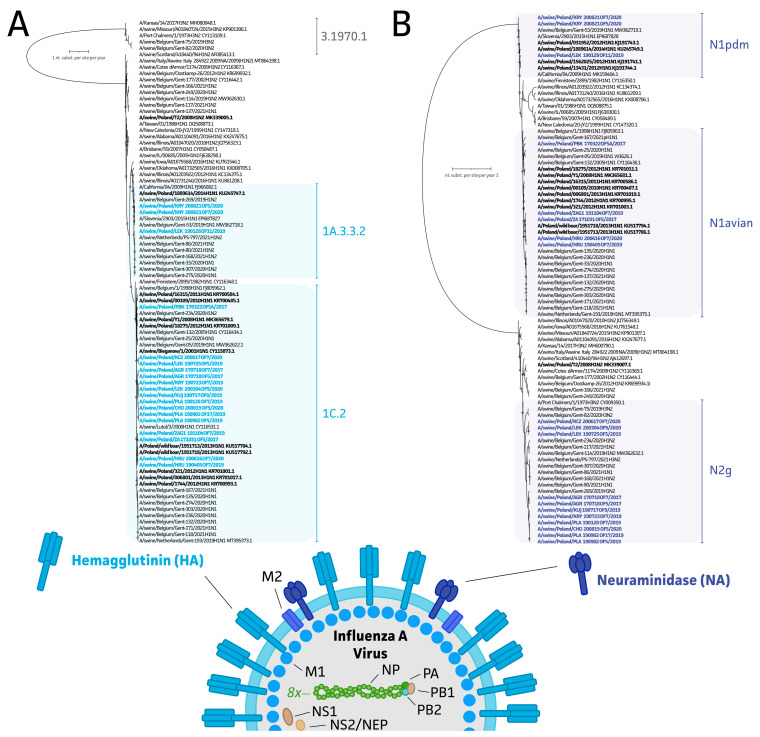
Maximum likelihood (ML) phylogenetic inference of swIAV hemagglutinin (HA) and neuraminidase (NA) gene segments. These are the surface glycoproteins of the swIAV virus particle as shown on the schematic representation of the IAV viral particle. (**A**) ML phylogenetic inference of the hemagglutinin gene segment with a representative European dataset to highlight the subclade classification of newly obtained Polish HA sequences (n = 19). Additionally, two HA sequences of near-complete genomes were included. HA classification based on the global H1 nomenclature system of Anderson et al. (2010) [4]. (**B**) NA-based phylogenetic inference to identify subclades of swIAV within the obtained Polish population (n = 19). Additionally, two NA sequences of near-complete genomes were included. Horizontal distances represent number of nucleotide substitutions per site per year. Only bootstrap values (1000 ultrafast UFBoot bootstraps) with less than 95% support are shown on the tree. Relevant swIAV strains were included in this tree, and available Polish strains and newly sequenced strains are highlighted in black and blue/purple, respectively.

The resulting (near-)complete swIAV sequences were used to study swIAV diversity across Polish farms between 2017 and 2020. As shown in Figure 3A, all strains (n = 19, representing 14 herds; 17 complete and 2 lacking the S2 IGC gene segment) showed an H1 subtype, of which 3 (15.8%) and 16 (84.2%) swIAV HA segments could be further classified into the 1A.3.3.2 and 1C.2 subclades, respectively, based on the global swine H1 nomenclature system [4]. When studying the NA segment in Figure 3B, 42.1% (8/19) of the strains belonged to the N1 subtype, with three strains showing an H1N1pdm09 origin and five originating from avian N1. All the other NA segments (11/19) belonged to the N2 subtype and N2g subclade (Figure 3B). To further determine potential reassorting events, the origin of all internal gene segment sequences was determined. As summarized in Table 1, 91.3% of the sequenced viruses had internal genes that originated from an H1N1pdm09 lineage. The two remaining strains showed a Eurasian avian origin. This allowed us to perform swIAV genotyping based on the schemes provided by Watson and colleagues (2015) and Hentritzi and colleagues (2021) [5,6]. Most of our strains were classified in the T genotype (11/19). This was followed by U (n = 4), P (n = 3), and A genotypes (n = 1). On a herd level this indicates that 58.3%, 25%, 16.7%, and 0.1% of all herds showed a T, U, P, or A genotype. Samples collected from the same farm in the same or consecutive year showed the circulation of the same swIAV subtype and lineage. This is exemplified by the subtypes from herds AGR (2017), HRU (2019 and 2020), and PLA (2019) (Table 1). Only in the herd with the KRY T genotype (H1N1pdm09 ICG with Eurasian avian and A/swine/Gent/1/1984-like H3N2 origin for the HA and NA segment, respectively) was identified in 2019, after which the P genotype (H1N1pdm09 subtype) was detected in two samples originating from 2020.

## 4. Discussion

The collection of oral fluid samples is a very easy and noninvasive procedure by which a large number of pigs present in the pen can be sampled. Many studies showed the usefulness of this material for PCR detection and nucleotide sequencing with the Sanger method. However, its usefulness for direct WGS has been rarely exploited. Some studies showed its use for WGS of PRRSV, porcine astrovirus, and PCV-3 [16,17]. Important to note is the fact that oral fluids are considered complex samples as compared to typical nasal or oropharyngeal swabs. The “dirty” matrix is composed of oral mucins, enzymes, antibodies, microbes, drug components, feed particles due to the passage of food, and potential contaminants from the environment (e.g., from ear biting and contact with stool). Hence, to fit with existing molecular protocols, adaptations are required to assure proper and accurate pathogen detection [33]. Thus, it is often assumed that the highly complex contents of oral fluids make such a material inappropriate for WGS.

In this work, a total of 34 oral fluids were collected from 19 different farms in Poland between 2017 and 2020. First, the impact of storage conditions on IAV RT-qPCR results was assessed. This was possible since the samples collected in 2017 were stored at −22 °C, after which they were transferred to long-term storage at −80 °C in 2019. Samples collected in 2019 and 2020 were immediately stored at −80 °C. Apparently, the primary storage of samples at −22 °C and the later transfer to −80 °C had a major impact on the observed swIAV detection as compared to the samples that were immediately stored at −80 °C (ΔCq of 6.3 (±4.9 SD) and 0.6 (±2.3 SD), respectively). Of note, even though two different RNA isolation and swIAV RT-qPCR approaches were used, the14 samples still showed comparable Cq values pre- and post-storage (ΔCq < 1.5). Whether the initial −22 °C storage or the transfer to −80 °C after two years impacted the RNA cannot be concluded from our results. Interestingly, a study by Foster and colleagues (2008) studied the impact of IAV detection in a complex matrix, showing a significant reduction in IAV detection in the presence of a fecal matrix and freeze/thawing, although their work focused on an RNA-stabilizing agent [34]. Indeed, one of our samples was classified as highly unpure and exhibited the biggest loss of swIAV detection after prolonged storage at −22 °C (ΔCq = 15.1). It is important to note that no correlation was made between the visual sample quality and reduction in swIAV viral loads detectable via RT-qPCR. This finding also has practical importance regarding the PCR detection of other viral pathogens, as it indicates that the visual evaluation of the oral fluid sample does not allow us to predict difficulties in the detection of a pathogen present there.

Since the SARS-CoV-2 pandemic started in 2020, extensive focus was put on investigating the impact of various conditions, including sample collection/type, transport, and storage conditions, on molecular diagnostic results and the interpretation of respiratory samples [35,36,37]. As shown in the Bayesian network meta-analysis of Hou et al., (2020), nasopharyngeal washes, mid-turbinate, and nasopharyngeal swabs were ranked highest as good sampling methods for the successful detection of a wide variety of respiratory pathogens [38]. These samplings methods were also considered best for the specific detection of IAV. Even though oral fluids were not implemented in this study, (nasal-)throat swabs were ranked as inferior sampling technologies for most of the viruses [38]. While the sampling method/type is important, standard procedures and the invasiveness of some methods might prevent their practical use (e.g., tracheobronchial swabs). As previously assessed, viral loads, expressed as Cq values, play an important role in the prediction of sequencing success. As shown for PRRSV, Cq values of 21 or lower were required to obtain complete genome sequences from oral fluids [17]. Here, complete swIAV genomes could be obtained from samples with Cq values of 27 or lower. Importantly, while short-read sequencing approaches did not allow us to accurately determine mixed infections (i.e., different strains), this is possible when using long-read sequencing [39]. Targeted approaches were also applied for other RNA viruses (e.g., astrovirus [18]) and DNA viruses, including PCV2 and PCV3 [16]. Whenever a broader screening is needed, metagenomics could also be applied. However, one should bear in mind that oral fluid composition is highly impacted by environmental contaminants [14,40,41,42,43].

Due to the complexity of oral fluid samples, two distinct targeted IAV sequencing protocols were evaluated [22,23]. While both protocols use the SuperScript™ III One-Step RT-PCR System with the Platinum™ Taq DNA Polymerase master mix (Invitrogen™), differences are present in the conserved primer sets and actual PCR thermocycling conditions (Figure 2A,B). The use of different conserved primers produces the first factor that impacts the final sequencing success. Method 1 exploits two conserved primers (Pan-IVA-1F_M13F and Pan-IVA-1R_M13R), which were designed to supplement a more extensive IAV WGS by using a wide variety of additional segment-specific primers for 454 pyrosequencing [44]. The protocol, as presented by King and colleagues (2020), was chosen due to its proven multiplexing potential on the MinION sequencer [22]. In our hands, this method did not deliver sufficient sequencing coverage for all segments across samples. With a sequencing success of only 32%, the protocol cannot be considered successful for targeted long-read nanopore sequencing from oral fluids. Here, the complexity of the oral fluid samples might have posed a problem, as King and colleagues’ results were obtained from egg-grown viral stocks, representing a “perfect” matrix with few impurities and an enriched population of (intact) IAV particles. Thus, our work emphasizes the importance to include real field samples, such as oral fluids, bronchoalveolar lavages, and nasal or oropharyngeal swabs, in the validation and implementation of new sequencing-based diagnostics approaches. Indeed, some samples (8/25) with higher viral loads showed the recovery of complete swIAV genomes when using method 1 (data not shown) [22]. Method 2, on the other hand, exploits three universal IAV primers (CommonA-Uni12G, CommonA-Uni12, and CommonA-Uni13G). These primers were first introduced by Watson and colleagues in 2013 to be used in next-generation, short-read Illumina sequencing to detect minor viral variants within a population [45]. Even though their amplification protocol used three individual RT-qPCR reactions, various adaptations to the protocol have been made over the years to deliver more cost-efficient IAV sequencing alternatives [23,46,47]. The protocol was shown to be compatible with third-generation MinION sequencing workflows in our work, as well as in other reports in the context of swIAV epidemiology and point-of-care testing [48,49,50,51].

A second difference between the two methods can be found with the PCR thermocycling condition. In method 1, a traditional (60 min at 55 °C) reverse transcription step is implemented, whereas an extended RT step is found in method 2. The latter consists of three individual RT incubation steps, in which the RT is performed at a temperature of 42 °C, 55 °C, and finally 60 °C for 15 min, 15 min, and 5 min, respectively [45]. As described for the SuperScript III enzyme (and others), the thermostability of the enzyme allows for a wider range of temperatures to be used. Tweaking the RT temperature allows us to define the stringency of primer annealing (e.g., CommonA primers). Furthermore, increased temperatures allow for higher cDNA yields for RNAs with secondary structures [52,53]. This is hypothesized to explain why HA and NA segments turn out to be the first ones to drop out of sequencing. As these segments are under the highest evolutionary pressure, they will represent the highest sequence varieties as compared to other segments. Indeed, in 80% of our samples, the NA segments could be sequenced using method 2, though only 24% of NA sequences could be generated with method 1. A second difference and potential contributor to sequencing success can be found in the ramping rate. Ramping rate is the speed at which the temperature is changed between two consecutive steps within a PCR thermocycling program. The importance of ramping rates has been previously shown for molecular diagnostics of members of the *Mycobacterium tuberculosis* complex in which correct ramping rates showed superior performance, and hence, more accurate diagnostics reporting [54]. By tweaking ramping rates, primers show improved annealing to regions within the RNA/DNA target with higher GC content and/or complex secondary structures, thus improving their RT and amplification [55]. Our results supported the use of method 2 to increase the sequencing success with oral fluids. To the best of our knowledge, this is the first study to evaluate the important impact of both RT and ramping rates on IAV one-step RT-PCR amplification and subsequent sequencing success. Thus, more focus should be put on these steps when developing RT-(q)PCR and sequencing-based tests for use in diagnostics. Altogether, these differences in method 2 are thought to result in a superior performance in the generation of (near-)complete swIAV genomes from oral fluids. The actual primers will probably impact the sequencing success through better and subtype-wide segment targeting, though the exact impact of these primers should be verified in future research [23,46,47]. Additionally, applying a range of RT temperatures and ramping rates during PCR amplification is thought to increase the chance of on-target binding of these primers in the presence of (highly abundant) other nucleic acids (i.e., (r)RNA from contaminating organisms or host in oral fluids) [33,52]. It is important to note that the generation of medium/high coverage does not necessarily imply that a complete segment sequence could be obtained. This is highly dependent on the overall read distribution across the different segments, as exemplified in Figure 2D, and can affect the final assembly (reference-based or *de novo*).

WGS-based molecular epidemiology and evolution of swIAV is of paramount importance from a veterinary and public health point of view. In Europe, the most recent coordinated effort on swIAV epidemiology was carried out under the European Surveillance Network for Influenza in Pigs 3 (ESNIP3) in 2010–2013 [5,56,57]. The project resulted in the generation of 231 complete and 12 incomplete genomes obtained from virus isolates from 2009 to 2013. Combined with the 47 genomes present in GenBank, the dataset consisted of 290 swIAV genomes from 14 European countries. As many as 23 different genotypes were identified that resulted from the reassortment of external glycoprotein-coding segments, as well as IGC [5]. A majority of those (67%) contained IGC derived from Eurasian avian-like H1N1, while a minority (27%) contained IGC from H1N1pdm09. Genotype A (H1avN1) was the most common (29% of all viruses) and genotype P (H1N1pdm09 of all viruses) was the third most common (12%). These two genotypes were represented by 9 and 11 Polish isolates [5]. In a more recent study on the genetic diversity of swIAV in Europe, the number of genotypes was expanded to 31 with 12 distinct hemagglutinin/neuraminidase combinations, which highlights the rapid evolution of this virus [6]. As many as 20 of those genotypes contained one or more segments derived from H1N1pdm09. Unfortunately, no viruses from Poland were isolated and analyzed in that study, despite 67 out of 524 (12.8%) submitted samples being PCR positive [6]. This underlines the importance of the present study that showed that properly stored oral fluid samples, with qPCR Cq values up to around 30, can serve as useful diagnostic material for well-optimized WGS with ONT, facilitating genetic swIAV surveillance protocols. Even though applying short-read, second-generation alternatives has been the “gold” standard, this also impacts sequencing turnaround time (i.e., real-time data availability), portability (i.e., readiness in outbreaks [49]), and cost per analysis. Costs associated with equipment and consumables represent, at minimum, a 20- and 2-fold increase, respectively [58].The study provided 19 complete and 4 near-complete Polish swIAV strains from 14 pig herds. Of the complete genomes the most prevalent was genotype T, which is a reassortment of H1av and Ghent-like H2 with IGC from H1pdm, that was found in 7 herds (58.3%). Interestingly, Henritzi et al. (2020) identified such a genotype in only 10 out of 233 isolates (4.3%) from 2015 to 2018. The other genotypes detected in Polish samples were A (H1avN1, one herd), P (H1N1pdm09, two herds) and U (H1avN1 with H1N1pdm09 IGC, three herds). The true range of the current diversity on Polish farms needs further study.

## 5. Conclusions

In summary, to obtain the highest swIAV nanopore sequencing success from oral fluids, we encourage keeping samples at 4 °C during transport and processing [59]. Oral fluids should be kept at 4 °C for a maximum of 24–48 h only. While instantaneous nucleic acid isolation favors swIAV detection and sequencing, aliquoted storage at −80 °C is encouraged for batch extraction and/or long-term storage. Extracted nucleic acids should be kept at −80 °C to assure swIAV RNA integrity is not affected, and repeated freeze–thawing should be prevented. Complete swIAV genome sequences can be obtained with the appropriate segment amplification protocol (e.g., Van Poelvoorde et al. (2021) [23]) after proper RT-qPCR swIAV assessment (Cq < 27). Altogether, this will allow for monitoring swIAV in an animal-friendly and cost-efficient manner.

## Figures and Tables

**Table 1:** Tabular overview of swIAV segment origin. Each segment of the complete (n = 17) and near-complete (n = 6) swIAV genomes were characterized to determine the origin of each swIAV segment. Classification and representation as adapted from Chepkwony et al. (2021), genotyping based on schemes described by Watson et al. (2015) and Henritzi et al. Asterisk (*) indicates segments with undefined nucleotide stretches (N > 20).

					Surface Genes			Internal Genes					
	Farm	Year	Subtype	Genotype	S4 HA	S6 NA		S1 PB2	S2 PB1	S3 PA	S5 NP	S7 M	S8 NS
A/swine/Poland/BY_171030_OF5/2017	BY	2017	HxNx	Und.				*					
A/swine/Poland/GNI_170321_OF9B/2017	GNI		HxN1	Und.									
A/swine/Poland/PBK_170322_OF5A/2017	PBK		H1N1	A									
A/swine/Poland/AGR_170718_OF5_2017	AGR		H1N2	T									
A/swine/Poland/AGR_170718_OF7/2017			H1N2	T									
A/swine/Poland/ZA_171031_OF5/2017	ZA		H1N1	U									
A/swine/Poland/HRU_190405_OF7/2019	HRU	2019	H1N1	U									
A/swine/Poland/HRU_200616_OF7/2020		2020	H1N1	U									
A/swine/Poland/KRY_190723_OF5/2019	KRY	2019	H1N2	T									
A/swine/Poland/KRY_200821_OF5/2020		2020	H1N1	P	*			*					
A/swine/Poland/KRY_200821_OF7/2020			H1N1	P									
A/swine/Poland/KUJ_190717_OF9/2019	KUJ	2019	H1N2	T									
A/swine/Poland/LEK_190725_OF5_2019	LEK		H1N2	T									
A/swine/Poland/LEK_190128_OF11/2019			H1N1	P									
A/swine/Poland/LEK_200304_OF9/2020		2020	H1N2	T									
A/swine/Poland/ZAG1_191104_OF7/2019	ZAG1	2019	H1N1	U									
A/swine/Poland/ZAG1_191104_OF11/2019			H1Nx	Und.									
A/swine/Poland/ZYW_191104_OF6b/2019	ZYW		H1Nx	Und.									
A/swine/Poland/PLA_190902_OF5/2019	PLA		H1N2	T									
A/swine/Poland/PLA_190902_OF17/2019			H1N2	T									
A/swine/Poland/PLA_190128_OF7/2019			H1N2	T				*					
A/swine/Poland/CHO_200819_OF5/2020	CHO	2020	H1N2	T									
A/swine/Poland/KCZ_200617_OF7/2020	KCZ		H1N2	T									
													
**European avian-like H1N1**													
**2009 pandemic H1N1**													
**Human-like H3N2**													
**Genetic information not available**													

## Data Availability

The data presented in this study are available upon request.

## Supplementary Materials

The following supporting information can be downloaded at: https://www.mdpi.com/article/10.3390/v15020435/s1, Table S1: Overview of samples, metadata, and sequencing information.
